# One template does not fit all: where next to improve hospital discharge communication to primary care?

**DOI:** 10.1017/S1463423625100327

**Published:** 2025-09-15

**Authors:** Nicholas Boddy, Joanne Reeve, Rachel A. Spencer, Anthony J. Avery

**Affiliations:** 1 Centre for Academic Primary Care, School of Medicine, University of Nottingham, Nottingham, UK; 2 National Institute for Health and Care Research (NIHR) Greater Manchester Patient Safety Research Collaboration (GM PSRC), The University of Manchester, Manchester, UK; 3 Academy of Primary Care, Allam Medical Building, Hull York Medical School, University of Hull, Hull, UK; 4 Warwick Medical School, University of Warwick, Coventry, UK

**Keywords:** interprofessional education, patient discharge summaries, primary healthcare, patient safety

## Abstract

Led by national policy, standardisation has enhanced hospital discharge communication to primary care over recent decades. However, discharge summary content standards and their corresponding templates can be over-relied on by authors, risking the exclusion of important contextual and explanatory information for patients with more complex care.

This information can be critical for GPs to deliver high quality, safe, and efficient post-discharge care, especially for this patient cohort which can be at higher risk of avoidable harm from suboptimal communication. Discharge summary authors can lack sufficient understanding of the recipient primary care perspective to mitigate this issue and communicate effectively through standardised letter templates. Strengthening this interprofessional understanding is an essential next step to improve discharge communication.

In response to this challenge, we propose the basis of a new framework of interprofessional discharge communication that accounts for the different paradigms of specialism and generalism and supports summary authors to tailor their content to the patient’s post-discharge care.

We call for the co-development of this framework through a programme of applied research, alongside the exploration of primary–secondary care interface learning communities as a vehicle for interprofessional education. These initiatives can serve to augment the current strengths of standardised discharge summaries and mitigate their limitations, maximising the quality, safety, and efficiency of post-discharge care. Progress in this field can benefit wider cross-interface communications and practice and assist the NHS integration agenda.

Standardisation of discharge summaries has benefitted interprofessional discharge communication practice over recent decades. This has been actively encouraged by national policy (Carpenter, 2008; NHS England, 2018) including by the Professional Records Standards Body’s latest e-discharge summary standard (Professional Records Standards Body, 2018). However, this strategy has also led to an over-reliance on a ‘one-template-fits-all’ approach, to the potential detriment of communication quality for patients with more complex care (Boddy *et al.*, 2021). Standardisation risks the exclusion of important contextual or explanatory details used by recipient GPs to deliver tailored expert generalist (Reeve, 2023) community care, an approach that is often misunderstood by hospital-based authors (Reeve, 2022). Although generative artificial intelligence (AI) has been mooted as the future of discharge summaries (Patel and Lam, 2023), it is unlikely to alleviate this issue, given that the problem relates to differing perspectives on communication quality between standardised and tailored healthcare. We will discuss that improving discharge communication to patients represents a separate challenge and distinguish this from the unresolved debate on how to further improve interprofessional discharge communication (Boddy *et al.*, 2021; The Professional Records Standards Body, 2023). In response to this debate, we will outline the opportunity for research to develop a new framework of interprofessional communication across care boundaries that explicitly highlights the different paradigms of care used and supports discharge communication to be tailored to individual need.

## How standardisation has improved service delivery

As the near-exclusive format of discharge communication to primary care in the NHS, discharge summaries hold a critical role in patient safety and quality of continuing care (Healthwatch England, 2017; Spencer *et al.*, 2018). The necessity of their timely delivery to primary care has been clearly highlighted (Kripalani *et al.*, 2007), and the electronic delivery of summaries within 24 hours of discharge is now a contractual obligation (NHS England, 2016). The quality of content is also vital (Patterson, 2008; Tandjung *et al.*, 2011; May-Miller *et al.*, 2015; Caleres *et al.*, 2018), and the last two decades of policy and improvement work have centred on setting information standards (Carpenter, 2008; Academy of Medical Royal Colleges, 2008; NHS Digital, 2017a, 2017b; Professional Records Standards Body, 2018) and using summary templates with corresponding headings to deliver them. In the UK, these standards are highly generic and designed to be applicable to any patient (Professional Records Standards Body, 2018), with quality typically measured by adherence levels (Hammad *et al.*, 2014; May-Miller *et al.*, 2015; O’Connor *et al.*, 2018; Scarfield *et al.*, 2022). Incomplete handovers can be associated with higher risks of medication errors (Bergkvist *et al.*, 2009), deterioration in chronic conditions (Humphries *et al.*, 2020), readmissions (Al-Damluji, 2008), and death (Kripalani *et al.*, 2007; Schwarz *et al.*, 2019; Humphries *et al.*, 2020); standards and templates aim to reduce these with specific guidance for authors. Higher compliance with standards has received positive feedback from GPs as end users both in the UK (May-Miller *et al.*, 2015; Weetman *et al.*, 2021) and internationally (Van Walraven *et al.*, 1998; Kripalani *et al.*, 2007; Dean *et al.*, 2016; Gilliam *et al.*, 2017).

## The limitations of standardising discharge summaries

Despite significant benefits, concerns have been raised about the suitability of these generic content standards to serve all patient cases and optimally drive the quality and utility of discharge summaries from all perspectives (Boddy *et al.*, 2021). Adhering to standards may not fully align with other validatory metrics of quality, such as the notions of a successful discharge summary (Weetman *et al.*, 2021) and the guarantee of episodic continuity of care(Braet et al., 2016). These highlight that standards do not fully address other factors such as clarity of language (Weetman *et al.*, 2021), document structure (Spencer *et al.*, 2019; Tesfaye *et al.*, 2023) (which is instead determined by local IT software capabilities), and the inclusion of tailored condition-specific information (Gusmeroli *et al.*, 2023) (such as creatinine trends in acute kidney injury). Broadening the requirements for discharge summaries to address these issues raises the challenges of deciding what is relevant to include within free text fields (Wimsett, Harper and Jones, 2014) such as the clinical narrative (Professional Records Standards Body, 2018), and how to use an appropriate degree of detail. GPs criticise summaries for missing relevant content (Yemm *et al.*, 2014) but also describe the negative effects of including excessive, irrelevant, non-tailored information (Hopcroft and Calveley, 2008; Mahfouz *et al.*, 2017; Chatterton *et al.*, 2024) which can hide important points and consume clinician time. This subjective challenge of deciding what information is relevant is compounded by the different perspectives on quality of primary and secondary care (Yemm *et al.*, 2014; Weetman *et al.*, 2021), meaning that quality of interprofessional communication has become difficult to consistently define (Wimsett *et al.,* 2014; Sorita *et al.*, 2021).

In combination, these issues undermine standardisation of discharge summaries as the panacea for improving interprofessional knowledge exchange at hospital discharge (Wimsett *et al.,* 2014; Boddy *et al.*, 2021), particularly given the increasing numbers of patients who have complex health problems such as multimorbidity (Faitna *et al.*, 2024), polypharmacy (Moriarty *et al.*, 2015), and multidisciplinary care (Stokes *et al.*, 2016). Patients with higher complexity are recognised to increase the difficulty of discharge communication: stressing the importance of adequate detail from the recipient primary care perspective and making it harder for hospital authors to curate the necessary level of detail (Boddy *et al.*, 2021). The risk of error can also increase (Boddy, 2019), and in the event of suboptimal communication, more complex patients may be at higher risk of avoidable harm (Das *et al.*, 2018). This is concerning, as standards and templates can become less supportive as complexity increases; the patient’s care may not ‘fit’ them (Boddy *et al.*, 2021). Higher complexity cases therefore expose an ‘Achilles heel’ in the standardised status quo, with potentially greater risks to patient safety and quality of continuing care.

## Using a purpose-driven approach to mitigate the limitations of standardisation

**Figure 1.**
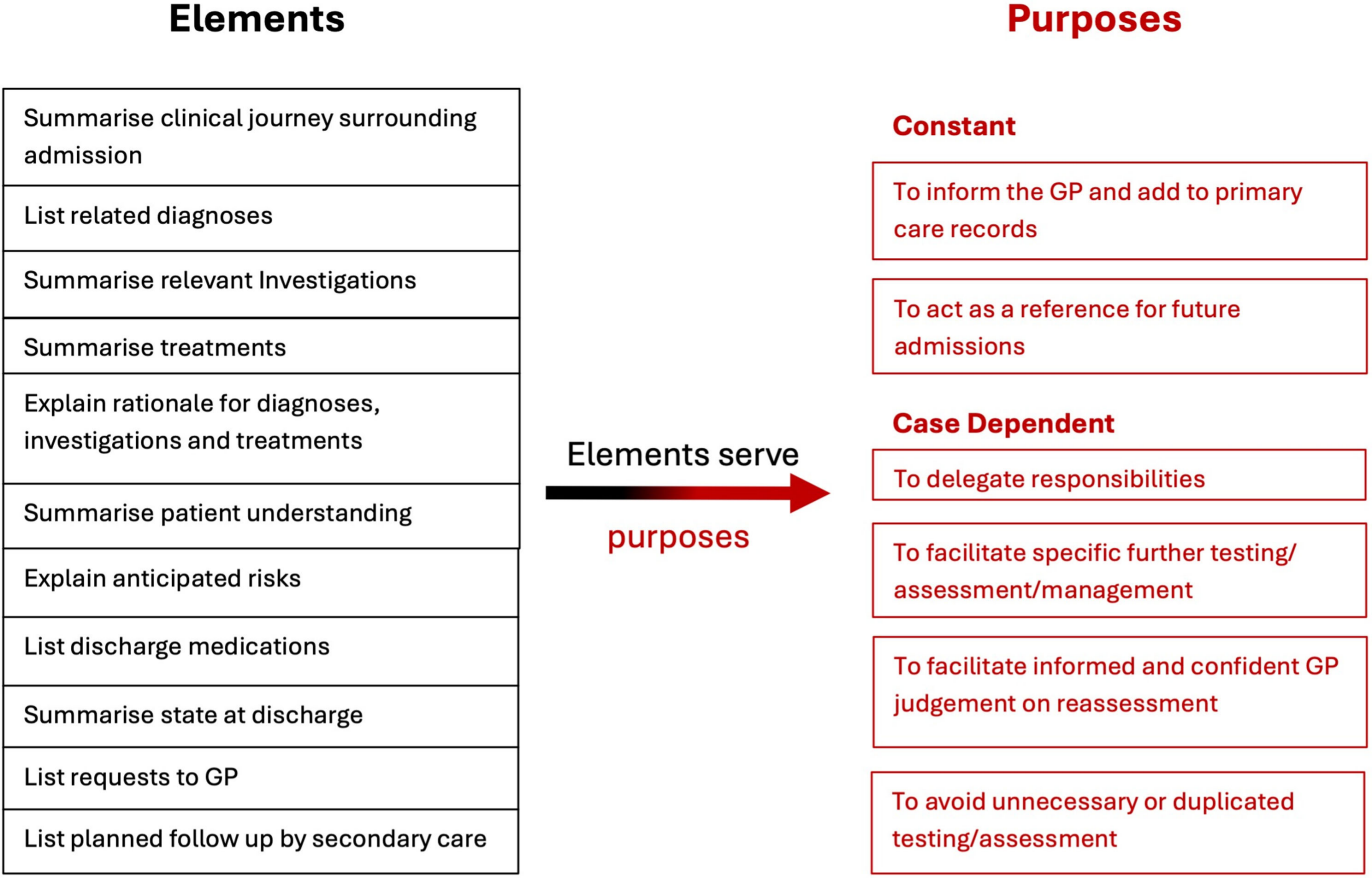
The purposes of interprofessional communication to primary care at discharge (adapted from Boddy, 2019 and Boddy *et al.*, 2021) Clinical Information elements within a discharge summary serve specific purposes. Some purposes are ‘constant’ and universally applicable, whilst others are dependent on the individual case.

**Figure 2.**
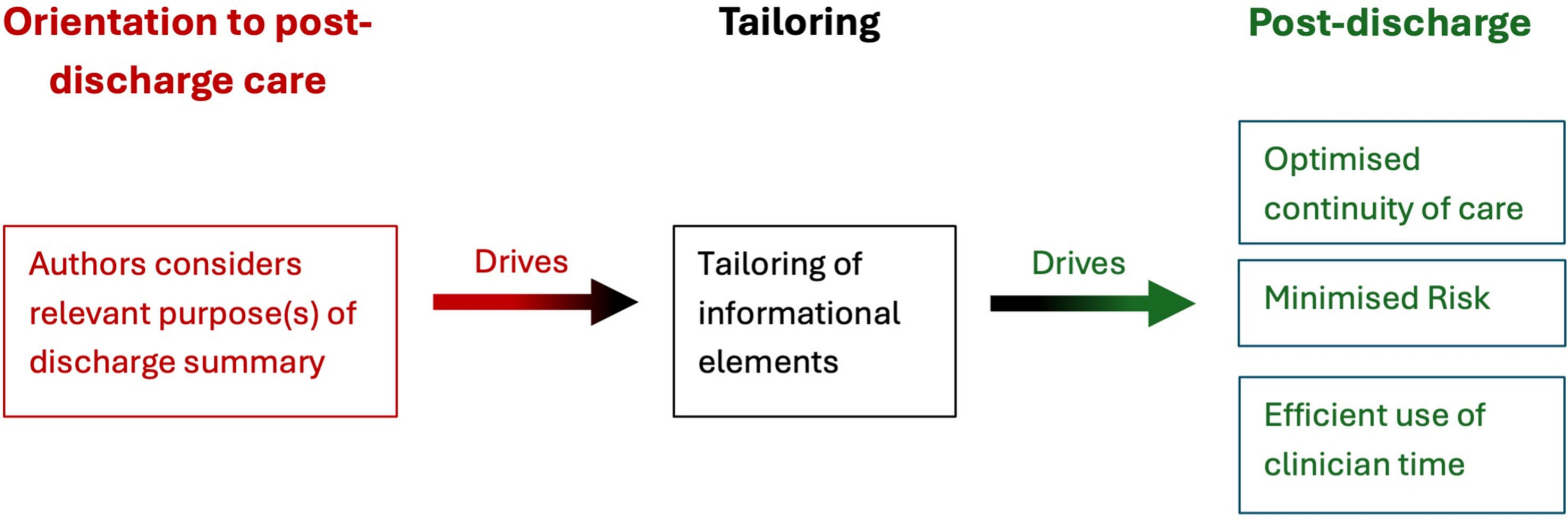
A driver diagram illustrating a ‘purpose-driven’ approach to interprofessional discharge communication (Adapted from Boddy, 2019 and Boddy *et al.*, 2021): By first considering the relevant purposes of the discharge summary for the individual patient, the author can tailor the detail of informational elements to serve post-discharge outcomes.

To mitigate the limitations of information standards and summary templates, it has been argued (Boddy, 2019; Boddy *et al.*, 2021) that discharge communication should become more tailored to the individual patient and orientated to their post-discharge care. To achieve this, a purpose-driven approach (Boddy *et al.*, 2021) has been proposed, where discharge summary authors are encouraged to look beyond the standardised template headings and focus more explicitly on the foreseeable purposes the document will serve. As Figure 1 illustrates, elements of information (such as those within the Professional Records Standards Body standards (Professional Records Standards Body, 2018)) will serve variable purposes. By considering the relevant purposes involved, authors can tailor the detail of each element to that task (Figure 2). A typical example of this would be providing details about fluid balance and the trajectory of diuretic dosing used during admission, to support a GP to review the patient’s diuretics post-discharge. Similarly, if asking a GP to review benzodiazepine use in a patient discharged to a care home with persistent signs of hyperactive delirium, details could be provided regarding the frequency and dosages required during the admission. Without considering these future post-discharge tasks, authors might consider such information to be irrelevant and consciously exclude it. The recipient GP would then be unable to take advantage of existing knowledge that could improve safety, reduce the risk of falls (if a previous diuretic or benzodiazepine dose was previously excessive), and maximise symptom control (if a previous dose was inadequate). Concisely presenting this information in a discharge summary would also be significantly more efficient for the GP to read, when compared to trawling through extensive shared medical records, which are used in areas such as Uppsala in Sweden (Tully *et al.*, 2013).

## Bringing theory to practice: the need to understand the recipient perspective

Focusing on purpose in this way is in keeping with broader communication theory such as Berlo’s Process of communication (Berlo, 1960), which indicates that purpose is central to effective communication. Building on the classical theories of Aristotle and Faculty Psychology, Berlo states that *‘the purpose of communication is to influence: to affect with intent’*. He further remarks that ‘*we can often lose sight of our purposes for communicating*’, to the detriment of efficacy. This underlines that if a discharge summary author completes the template headings, without consideration of the purposes the document will serve for the individual patient, then quality is likely to suffer.

Berlo’s theory also highlights the equal importance of the recipient’s purpose in a communication process, and that if the purpose of the ‘receiver’ is not compatible with that of the ‘source’ (i.e. the author), then communication breaks down. The different perspectives and purposes that primary care recipients and secondary care authors may bring to the discharge communication process are analogous to this principle, as demonstrated by a recent study that found GPs and hospital doctors disagreed whether a discharge summary was successful in 44% of cases (Weetman *et al.*, 2021). Neither perspective should be considered ‘wrong’, but in line with Berlo’s theory, mutual compatibility of purpose must be maintained for communication to be effective.

In the context of improving discharge communication, author understanding of the recipient GP perspective has already been described as a barrier (Yemm *et al.*, 2014). Hospital doctors have been shown to lack insight into the nuances and practicalities of community care (Wills *et al.*, 2011; Yemm *et al.*, 2014; Jones *et al.*, 2015; Kable *et al.*, 2018), meaning that if a GP asks a hospital author to ‘*imagine you’re me*’(Chatterton *et al.*, 2024) when completing a summary, they may not be able to do so accurately. Anticipating the purposes that GPs will use information for may therefore be very difficult. Natural opportunities to improve this interprofessional understanding are significantly limited by the largely one-way structure of NHS discharge communication system, where hospital discharge summary authors very rarely receive feedback from their recipients, if at all (Boddy *et al.*, 2021). Existing national standards (Professional Records Standards Body, 2018) and educational packages such as the Royal College of Physicians learning resource (The Royal College of Physicians, 2018) do not directly address the risk of communicating with ‘misaligned purposes’, further compounding the issue.

## The patient perspective

Discharge summaries should also be offered to patients (Weetman *et al.*, 2021; NHS, 2023). A recent realist evaluation found convincing overall benefits of doing this, with clear improvement of patient health literacy, satisfaction, and empowerment, even if technical information is used (Weetman *et al.*, 2019, 2020; Weetman *et al.*, 2021). Such technical information may be incomprehensible to patients and their carers (Harris *et al.*, 2018), but its use was regarded as an ‘inherent need’ by GPs, and hospital doctors expressed that oversimplification can reduce quality from the interprofessional perspective (Weetman *et al.*, 2020). This indicates that patients and professionals are likely to have significant differences in their needs from discharge summaries and in the purposes that they use them for. The important task of meeting these potentially contrasting needs with a single summary may therefore be extremely difficult to consistently achieve. This may be particularly challenging for patients who have complex care, when technical explanations and terminology may become even more important for GPs to receive. The tailoring of purpose-driven discharge summaries should therefore extend to include the technical level of language needed by professionals. This may ultimately necessitate the production of separate patient-facing and GP-facing summaries. Whilst time-consuming, the benefits of this have been explored (Lin *et al.*, 2012, 2014; Weetman *et al.*, 2021), and in future, the use of generative AI to ‘translate’ technical discharge summaries into patient-friendly language (Kim *et al.*, 2024; Zaretsky *et al.*, 2024) may significantly reduce the additional workload. However, detail on the delivery of patient-focused discharge communication is beyond the scope of this article, and extensive dedicated research and improvement work continues to be undertaken by other research teams (Becker *et al.*, 2021; Spencer and Singh, 2021; Spencer *et al.,* 2025).

## How to understand the recipient generalist paradigm

**Figure 3.**
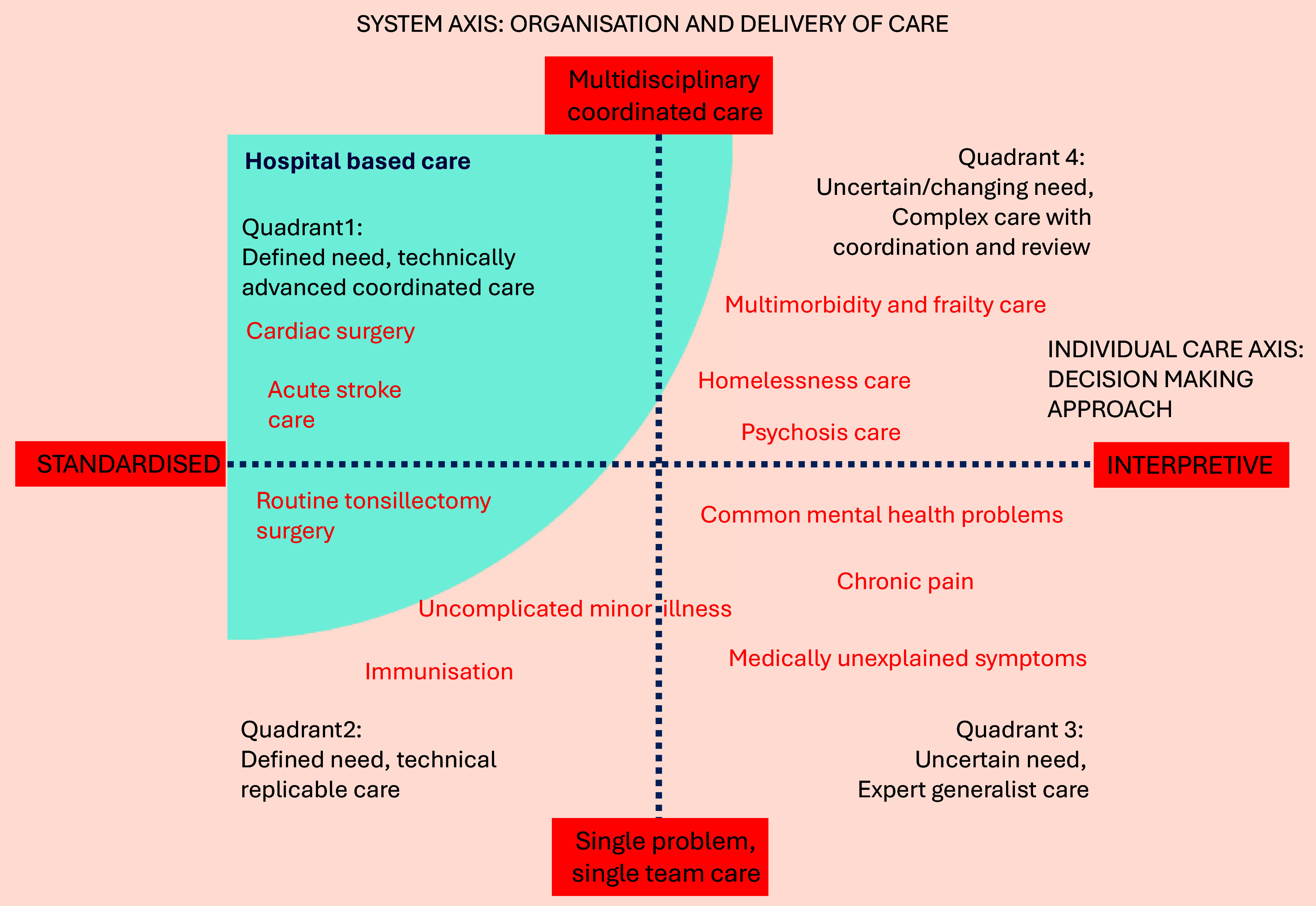
The United Model of Generalism (adapted from Reeve and Byng, 2017), illustrating how the differing needs of patients may benefit from the traditionally different decision-making approaches of generalist and specialist care. Hospital discharge can represent a change to an interpretive decision-making approach, which may require greater contextual information within discharge communication.

To further improve interprofessional discharge communication, the emergent question is: how can hospital authors better understand the recipient primary care perspective? We argue this should start with an understanding of the traditionally different approaches of generalism and specialism. There are common misconceptions of generalism, such as GPs operating as ‘guideline machines’(Smith *et al.*, 2021) and ‘jacks of all trades’(Reeve, 2023). These are now increasingly dispelled by a recent redefinition of ‘expert medical generalism’: the ability to work with a patient to tailor a management plan to their individual needs as a ‘whole person’, in an interpretive manner (Reeve, 2010, 2023). This involves a different approach to decision-making, where ‘best-fit’ management plans are tailored and account for contextual details, in order to work beyond the limitations of clinical guidelines for individual patients, especially those with complex care needs. This process, also referred to as ‘knowledge work’ (Reilly *et al.*, 2021), contributes significant value to the quality and safety of care provision. It is likely to be highly beneficial for discharge summary authors to appreciate the nature of this approach when trying to understand the purposes a recipient GP will use the content of the summary for, and particularly how they may handle complex care. These concepts are illustrated by the united model of generalism (Reeve and Byng, 2017) in Figure 3, which was devised to demonstrate how different care needs can require the differing approaches of generalist and specialist care, as well differing levels of multidisciplinary care. It also shows how discharged patients’ needs may cross over from needing standardised care in hospital, to needing interpretive expert generalist care predominantly in the community: a change in the paradigm of care and decision-making, as has been recognised more generally at the primary–secondary care interface (Johnston and Bennett, 2019). The model is therefore an ideal start point for discharge summary authors to conceptualise when including greater contextual detail may become of greater value after discharge, when compared to inpatient care.

Patients receiving standardised specialist care for single problems (quadrant 2), such as a young adult with no past medical history admitted for an elective tonsillectomy, are likely to have little need for expert generalist interpretive care after discharge. There would be no obvious need for additional contextual details to be included, meaning this type of standardised care scenario can benefit maximally from the existing style of information standards. However, patients with ‘uncertain and changing complex care needs, requiring coordination and review’ (quadrant 4) are likely to benefit from expert generalist care after discharge, and a higher level of contextual, narrative, and explanatory detail is likely to be beneficial for GPs to maintain quality and safety. Examples might include details regarding chronic pain medication discussions with the patient, that the GP might later build on in future decisions, or the rationale for anticoagulation decisions in patients with a recent increase in falls risk, that might later evolve. If authors can use this type of framework to tailor the level of detail (Figure 2) to where it is helpful and not a hindrance, the benefits of information standards can be embraced whilst their limitations are simultaneously mitigated. Without this mitigation, the increasing uptake of standardisation being encouraged (The Professional Records Standards Body, 2023) is unlikely to improve discharge communication, particularly for patients whose care relates to quadrants 3 and 4.

## Next steps: actions for improvement

We discuss three key opportunities to increase secondary care teams’ understanding of the community-based expert generalist paradigm and to improve discharge communication:

### Improved support for authors

In order to translate these principles into practice, enhanced author guidance documents and bespoke teaching programmes that encourage greater interdisciplinary understanding will be required. As a basis for these, we call for the development of an expanded framework of interprofessional discharge communication co-designed by both primary and secondary care that takes full account of both perspectives and explicitly addresses the differences in paradigms of care. To develop this type of framework, research is needed to identify case characteristics that can prospectively indicate the need for interpretive care after discharge. The united model of generalism offers some starting points, but these will need significant development for the context of hospital discharge. However, creating discrete groupings or rigid scoring systems must be avoided as these could lead to the same limitation of information standards: distracting the author from the nuances of an individual case. Exploratory research is also needed to better understand how GPs use their generalist expertise in the post-discharge care phase, and how contextual information assists them to do so, in order refine the most important qualities of interprofessional communication. These could be used to inform adaptations to discharge summary templates and new quality metrics that evaluate far more than the fulfilment of information standards or simple notions such as ‘success’. In turn, these measures could underpin formal teaching programmes for discharge summary authors and act as a basis for formative feedback audits. In parallel, these initiatives will benefit from a supportive culture for discharge summary authors. Given over 90 % of discharge summaries can be authored by resident doctors in their first two years after qualification (Cresswell *et al.*, 2015; Shivji *et al.*, 2015; Bodagh and Farooqi, 2017), their senior clinical colleagues, who may have greater experience of the healthcare system, can play an important role in advising and feeding back on content, as well as ensuring discharge communication is appropriately valued (Boddy *et al.*, 2021). This cultural support will be particularly important given the intense time pressures and competing demands that authors face (Hesselink *et al.*, 2012; Wohlauer, 2012; Kable *et al.*, 2018).

### Direct exposure to reciprocal perspectives

Alongside greater support for authors, further direct exposure to general practice during postgraduate training pathways could provide rich osmotic learning experiences of the expert generalist paradigm. Additional primary care placements and reciprocal visiting schemes such as the ‘learning together’ programme (Macaulay *et al.*, 2013) for London paediatric and GP trainees could be vehicles for this form of learning, if promoted by educational policy. Other approaches to primary–secondary care interface education such as learning communities may also be of benefit. These are well established in other educational sectors and are designed as small groups of peers who meet to discuss, reflect, and share their professional judgements in a ‘safe space’ (Wilson and Lowe, 2019), with a particular focus on uncertainties and the development of shared ‘practical wisdom’. Similar types of groups are already common within specialities or departments, and groups such as safeguarding ‘peer-review’ meetings may be formally embedded. However, cross-interface groups (Janssen *et al.*, 2023) are likely to be novel to most health professionals and may offer particular benefits for communication relating to cases with more interpretive care and undefined needs. With an explicit focus on the different paradigms of care used across the health system, action research could be used to pilot and evaluate the potential impact of cross-interface learning communities (Spicer and Roberts, 2020) on discharge communication.

### Extending standards for specific conditions

If authors can use improved interdisciplinary understanding to manage standardised templates effectively, there is scope for research to extend the existing concept of information standards by developing key content lists for specific conditions. Some hospital departments already informally devise their own sub-templates to use within generic templates to this effect, and electronic health records systems are already able to dynamically insert bespoke fields when diagnoses are present, such as target oxygen saturations for patients with COPD. This could be of particular benefit to patients with more standardised aspects to their care (quadrant 2). In future, this capability could be supported by generative AI and expanded to a vast array of conditions, if regulated by extended national standards. Whilst generative AI has been shown to be able to produce discharge summaries with high fidelity to standards (Clough *et al.*, 2024), conversely it has been shown to lack the ability to deduce clinical rationale from clinical records (Ando *et al.*, 2022). It is therefore likely that including explanatory and contextual information in discharge communication will remain a human task and continue to rely on hospital authors’ understanding of the primary care perspective. As generative AI technology inevitably progresses, education of discharge summary authors will have to iteratively adapt to ensure that these potentially missing aspects are routinely considered, whilst maximising the benefits of increased accuracy and efficiency. This should be underpinned by research that evaluates artificially generated summaries with respect to contextual and explanatory information, and in relation to the of post-discharge care type (or quadrant of Figure 3) involved.

## Conclusion: benefits beyond discharge

To further improve, discharge communication and its related policies must evolve beyond the limitations of generic standards and templates. We propose a paradigm shift to purpose-driven summaries, rooted in a change in interprofessional understanding and practice. This can be achieved through the development of improved support for authors, experiential learning, and communities of practice. There will clearly be implementation challenges to overcome, most notably in terms of time and resource, as well as the historic cultural divide between primary and secondary care that these measures seek to bridge. However, any necessary organisational and technological changes, such as to educational pathways and IT software, should benefit cross-interface communication more widely and assist the NHS response to its integration agenda (Department of Health, 2012, 2022), further strengthening the case to pursue this body of work. Progress in this field will also contribute to greater understanding of what is required to work towards Lord Darzi’s proposed ‘left shift’ in patient care (Darzi, 2024) to the community.
